# Multi-Sensor Person Following in Low-Visibility Scenarios

**DOI:** 10.3390/s101210953

**Published:** 2010-12-03

**Authors:** Jorge Sales, Raúl Marín, Enric Cervera, Sergio Rodríguez, Javier Pérez

**Affiliations:** Department of Computer Science and Engineering, Jaume I University, Vicent Sos Baynat, s/n, 12071 Castellón, Spain; E-Mails: rmarin@icc.uji.es (R.M.); ecervera@icc.uji.es (E.C.); serodrig@sg.uji.es (S.R.); al088453@alumail.uji.es (J.P.)

**Keywords:** low-visibility, smoke, risky environment, laser rangefinder, ultrasound, TDoA

## Abstract

Person following with mobile robots has traditionally been an important research topic. It has been solved, in most cases, by the use of machine vision or laser rangefinders. In some special circumstances, such as a smoky environment, the use of optical sensors is not a good solution. This paper proposes and compares alternative sensors and methods to perform a person following in low visibility conditions, such as smoky environments in firefighting scenarios. The use of laser rangefinder and sonar sensors is proposed in combination with a vision system that can determine the amount of smoke in the environment. The smoke detection algorithm provides the robot with the ability to use a different combination of sensors to perform robot navigation and person following depending on the visibility in the environment.

## 1. Introduction

**Figure 1.**
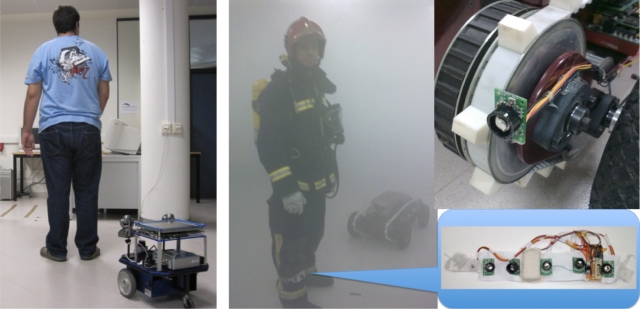
Robot following a human using computer vision (left). Robot following a firefighter using ultrasound and radio sensors. An ultrasound ring transmitter is attached to the firefighter’s leg. The robot can follow the firefighter using the received signals (right).

Person following performed by a robot (see Figure 1) has traditionally been an important research topic in the machine vision area. It is natural to imagine that mobile robots of the future, especially those operating in public places, will be expected to have this skill. As an example, in [1], an efficient person tracking algorithm for a vision-based mobile robot using two independently moving cameras is presented. With this approach it is possible to carry out real-time person following in indoor environments. This and other works described in next section have in common the assumption of good visibility and has been tested mainly in indoor environments.

In real situations it is not feasible to assume good visibility. The use of multiple sensors for acquiring information from the environment is proposed in this paper for adapting to each particular situation. The use of laser rangefinder and sonar sensors is proposed in combination with a vision system that is able to determine the degree of visibility in the environment. It is likely that we will soon see robotic assistants appear in our households, offices, hospitals, shopping malls, and other human-populated environments. Because these robots interact primarily with the general public, it is important that human-robot interaction is intuitive and socially acceptable.

In the next section, other previous works related to person following are described. Then, in Section 3, a multiple sensor approach is proposed, including the use of a camera in order to determine the degree of visibility of the environment (Section 4). In Section 5, different person following methods are explained and compared (Section 6). Finally, the conclusion section discusses and proposes the use of the described multi-sensor system.

## 2. Review of Developed Systems

Some of the most relevant previous works that have tried to solve the person following problem are described in this section. For example, in [2], authors describe a robot with following function and returning function using a monocular camera. A different human tracking method using template matching in range data obtained from a laser rangefinder (LRF) is described in [3]. The block matching is performed using stored templates, which correspond to appearances of human legs. In [4], the authors use laser scans and filtering techniques for tracking moving objects and persons.

In [5], the authors use a laser-based person-tracking method and two different approaches to person-following: direction-following and path-following, and combination of both methods is proposed: a hybrid approach, with the robot automatically selecting which method to use.

A robot tracking system based on camera and light-emitting device is used in [6]. Their purpose is to develop an autonomous mobile robot which can follow a human being. The robot with a camera looks at a human having a light-emitting device. The device consists of two LEDs. The robot can know the position of the human from the distance between two LEDs and the direction from the position of the LED on the captured image.

In [7] a double sensor system is used to perform person following by a robot. The robot can accompany a person using vision based target detection and avoid obstacles with ultrasonic sensors while following the person.

All of these approaches make use of different sensors to perform person following but assume good visibility conditions. Robots used for rescue operations like the ones performed by firefighters must be able to navigate in smoke-filled environments (refer to the EU GUARDIANS project [8]). The aim of this work has been to develop a multi-sensor system, being able to follow a person maximizing its reliability and adapting to the visibility conditions. In the next section, a multiple sensor approach is described.

## 3. Multiple Sensor Approach in Low-Visibility Scenarios

**Figure 2.**
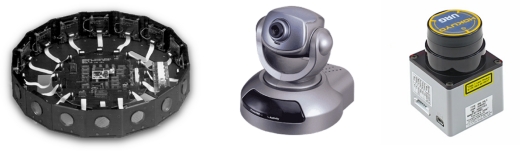
Sonar ring of sensors widely used for indoor mobile robots (left), Vivotek wireless pan-tilt camera (center), and Hokuyo URG-04LX laser range-finder typically used in robotics for navigation and localization (right).

Multiple sensors are traditionally used in robotics to allow the robots to navigate (see Figure 2). Some of them are laser rangefinders (LRF), ultrasound, computer vision, *etc*. The laser sensor has been widely used, both for navigation and simultaneous localization and mapping (SLAM) [9]. Their use is appropriate for most of the environments and provides a good resolution and precision compared to ultrasonic sensors.

In [10], authors study two 2D LRF commonly used in mobile robotics: the Sick LMS200 [11] and the Hokuyo URG-04LX [12]. In his study, those laser sensors were tested in extreme environmental conditions, particularly relevant for firefighting applications. Their conclusion is that in optimal conditions, namely good visibility, no interfering sources, and surfaces with good reflectivity in the sensor direction, LRFs are excellent range sensors, particularly after warming-up, in terms of linearity and accuracy. But in adverse environments, when the previous conditions are not met, LRFs provide erroneous or saturated outputs, becoming unusable as a range sensor for robotics [10]. One additional problem when using LRF for person following is that it is not easy to analyze the different patterns in order to distinguish among walls, objects or persons. Pattern analysis and quite a lot of processing must be done in order to identify a person walking and follow that person [13]. As a conclusion, the widely used laser rangefinder sensor in the field of mobile robotics should be complemented with the use of other sensors in the cases where there are no good visibility conditions, or it is necessary to carry out some kind of object or pattern recognition.

**Figure 3.**
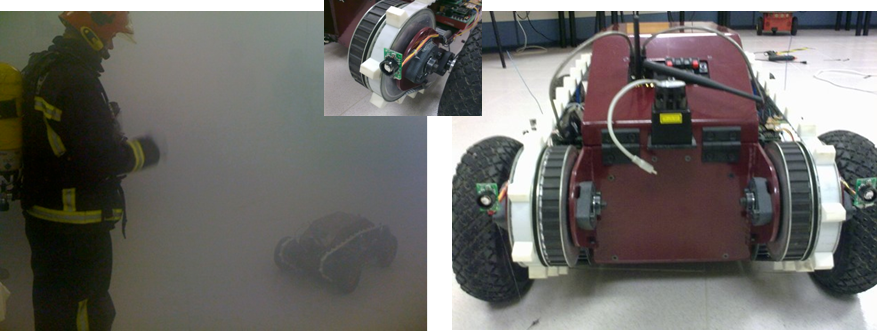
Guardian robot following a firefighter using a combination of radio and ultrasonic sensors.

The use of ultrasound sensors in alternative solution to LRF. Their performance is much lower than laser, but in some circumstances, like in smoke environments, they can provide a good solution (see Figure 3). In [14], the use of radio and ultrasound signals are used as a feasible alternative to robot localization.

Cameras can also provide a lot of information, but most of the existing works rely on the use of special landmarks or patterns, and good visibility conditions. The use of the camera is proposed in this article to determine the amount of smoke in the environment, allowing the multi-sensor robot to decide which sensor to use in each circumstance.

## 4. Detecting Smoke Intensity in the Environment

In order to decide whether the information coming from laser range finder sensors can be reliable or not, a method for determining the amount of smoke in the environment is proposed. Commercial smoke detectors typically used to detect fire are not usable for this purpose. Among the reasons we should emphasize that: (i) they only provide a simple on/off signal indicating the detection of smoke, and (ii) they can only warn of the presence of smoke if the smoke reaches the sensor, but can not warn of the presence of smoke at a distance from the sensor.

### 4.1. Proposed Smoke Detection Method

The proposed method uses a video camera (Vivotek PT3122 pan-tilt camera [15]) and is based on the smoke-detection method described in [16]. In that work, the authors propose a smoke-detection method for early fire-alarming system based on video processing. Their basic strategy of smoke-pixel judgment is composed of two decision rules: a chromaticity-based static decision rule and a diffusion-based dynamic characteristic decision rule. The chromatic decision rule is deduced by grayish color of smoke and dynamic decision rule is dependent on the spreading attributes of smoke.

Our implementation is based partially in the algorithm proposed in [16]. The method uses a reference image of the environment without smoke. Then, the reference image is transformed into HSI color model and we obtain the S plane (saturation plane) which is the plane containing the saturation of the image. When the environment contains smoke, the new image obtained under this circumstance is analyzed, using the method that can be described as follows:

- Extract the new image S component (Saturation) from its HSI color model.
- Calculate absolute difference between S components of reference image and new image.
- Get the binary image of the difference.
- For each positive pixel in the resulting binary image, use the following rules to verify if the pixel of the new image can be considered smoke.
  **–** rule_1 : R ± *α*_1_ = G ± *α*_2_ = B ± *α*_3_ (the three R, G and B components are similar, with 15≤ *α_i_* ≤20)
  **–** rule_2 : L_1_ ≤ I ≤ L_2_ (the Intensity component ranges from light-gray L_1_ and L_2_ values)
  **–** rule_3 : D_1_ ≤ I ≤ D_2_ (the Intensity component ranges from dark-gray D_1_ and D_2_ values)
  **–** If (rule_1) AND [ (rule_2) OR (rule_3)]: smoke pixel

### 4.2. Assessment of Laser Range Finder and Ultrasound Sensors in Smoke

**Figure 4.**
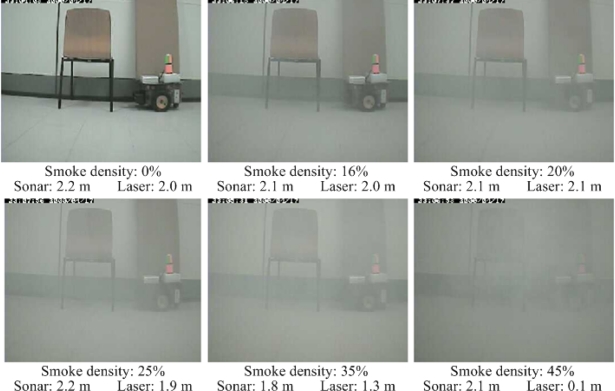
Sequence of images while increasing the smoke density.

In the described smoke circumstances, laser and ultrasound performance have been assessed. Figure 4, shows a sequence of captured images while increasing the smoke density. Each image is rated by the smoke detection algorithm from 0% to 45% of density. The assessment is performed by recording the distance value obtained from both laser rangefinder Hokuyo URG-04LX [12] and a sonar ring of eight MaxSonar EZ1 from MaxBotix [17].

The experimental procedure in order to assess the reliability of laser and ultrasound sensors is described next. It was performed indoors, in a 14 m^2^ room with 56 m^3^ volume. The room was well illuminated with fluorescent lights. To produce an increasing amount of artificial smoke, a commercial smoke machine using glycol-based fog juice was used.

**Figure 5.**
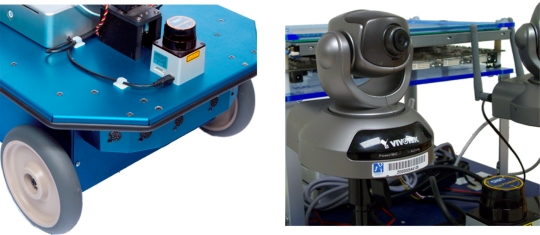
Erratic Videre Mobile platform equipped with Sonar ring of eight MaxSonar EZ1 and Hokuyo URG-04LX laser range-finder (left) and a Vivotek pan-tilt camera (right).

The robot platform (see Figure 5), an Erratic Videre mobile platform [18], is used for that purpose. The robot is placed 2 m away from the scene (a distance in the working range of the laser), and the measurements from the laser and sonar device are logged while increasing the smoke density. In Figure 4, for each smoke density, it is indicated also the distance measured by both sensors.

**Figure 6.**
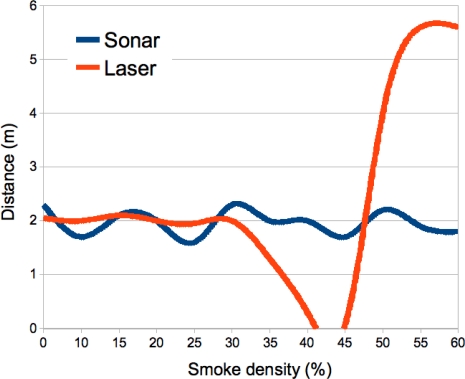
Sonar and Laser performance with increasing smoke density.

In Figure 6, the measured data obtained from both sensors is plotted while increasing the smoke in the scene. It can be seen that the sonar measurements are roughly stable around 2 m, independent of the amount of smoke in the scene. In the case of the laser, when the amount of smoke is less than 30% the measure is more accurate and stable than the sonar measure.

When the percentages of smoke are between 30% and 40%, a drastic reduction can be seen in the distance to the object, thus obtaining an error of this value. Finally, when the smoke density passes 45%, the laser is not able to provide a distance measurement. Since the laser beam is scattered by the amount of smoke particles, no reflection signal is detected by the sensor, thus a maximum distance of 5.6 m is assumed, as defined in the sensor settings.

The proposal, then, is to make the decision about using information coming from laser or ultrasound ring, based on the smoke density determined by the camera and algorithm used. A fuzzy logic algorithm is to be developed in future research work.

## 5. Person Following with Different Sensors and Experimental Results

In order to assess the performance of different sensors and algorithms to perform person following, several experiments have been developed. The three developed methods are: (i) Sonar Ring following, (ii) Laser Range Finder following and (iii) Sonar TDoA (time difference of arrival) following. The first one is based uniquely on ultrasound technology and the performance is not as good as the other methods. The second method uses a laser range-finder sensor in order to detect the legs of the person and provides better results. Finally, the third method uses a previous work based on a combination of radio and ultrasonic sensors to measure distances and carries out a follow strategy that provides good results even in low visibility conditions.

**Figure 7.**
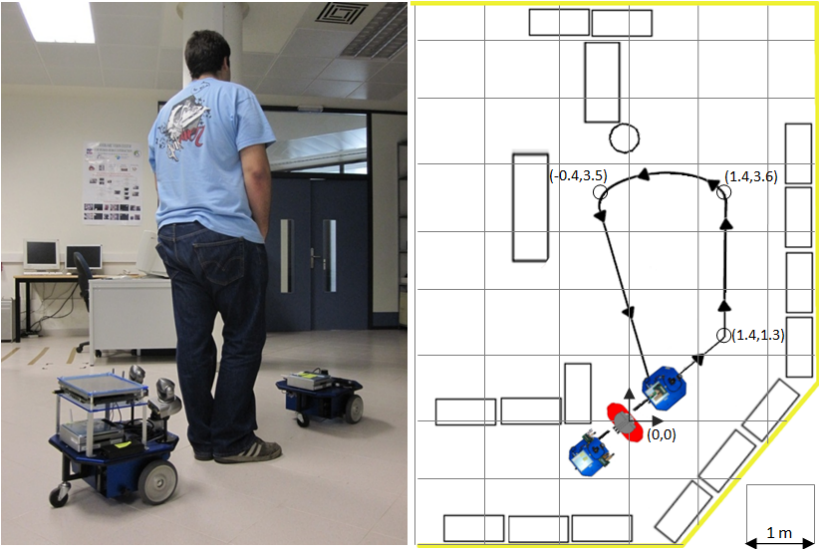
Person Following with different sensors. Robot formation (left). Predefined closed path with 3 left curves, with a detail of the coordinates of the trajectory (right).

To compare the different approaches, an indoors path was designed (see Figure 7). The details of the path measures are shown in Figure 7 right. The test consisted of a squad formed by:

- a former robot, following a predefined close loop path, at constant velocity,
- a human following the first robot, and
- a robot following the human.

The idea of this formation is to always maintain a constant speed and constant path. Also, the path contains 3 left curves, each curve sharper than the previous. The aim of the three sharp turns is to test the ability of each system to follow the human, even in tricky situations.

The robot platforms used in this experimental section (both the leader and the follower) are Erratic Videre mobile platforms [18] (see Figure 7 left) equipped with an embedded PC computer. The leader robot just executes a program to follow the predefined path with accuracy at the specified velocity. The follower robot integrates all the different sensors used in each method, and further explanation is given in the corresponding subsections.

To compare the performance of each method, 5 trials at different 4 velocities has been done for each following method. These three methods are described below in detail.

### 5.1. Sonar Ring Following

**Figure 8.**
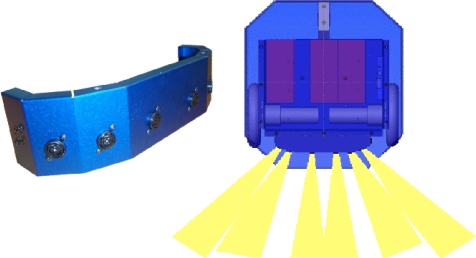
MaxSonar EZ1 ultrasound ring installed in the front of the Erratic Videre robot platform (left). Top view of the Erratic Videre mobile platform, showing the ultrasound pattern beam (right).

The initial person following configuration uses the built-in ultrasound ring (MaxSonar EZ1 from MaxBotix [17]) of the Erratic Videre mobile platform [18] (see Figure 8). The robot executes an algorithm that tries to follow a person by using the received information from the sonar ring. The algorithm assumes that the person to be followed is in front of the robot when several front sensors measure a short distance (50 cm in this experiment). After this initialization, the algorithm calculates the robot movements in order to maintain the followed person at the predefined distance, using the measures obtained by all the sonar sensors in the ring.

### 5.2. Laser Range Finder Following

**Figure 9.**
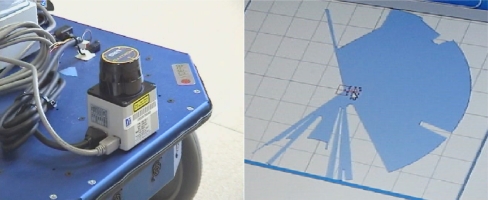
Hokuyo URG-04LX laser rangefinder sensor used for pattern tracking and following (left). Beam pattern emitted by the sensor and example of range measurement done in a complete 240° scan (right).

In this configuration, a Hokuyo URG-04LX laser range-finder [12] is used to detect the legs of the person and carries out a follow strategy using a pattern detection [13] (see Figure 9 left). This sensor emits a beam pattern that covers 240° in each scan (see Figure 9 right). Its maximum range is 4 meters, and provides an accuracy of +/− 10 mm, with an angular resolution of 0.36 degrees and a maximum scan rate of 10 Hz.

The algorithm assumes that only one person will be in the field of view of the follower robot (Erratic Videre mobile platform [18]). Using this precondition, the algorithm searches for an object of a certain configurable width and in an specific range of view and distance from the robot. This object will be the person as no other object will be in this initial range.

To know the object’s width, a linear search is performed at both sides of the object centroid. It is assumed that a point belongs to the object if it is inside certain threshold of distance (δ) of the object’s centroid from the robot (the laser reading value) and not far from this centroid in terms of the angle of the reading (ɛ). Those two parameters provide the dimensions of a rectangle which contains the shape suitable for tracking. Once the algorithm is initialized with the proper legs shape, it is able to track the patterns and command the robot perform a person following (see [13] for further details on the implemented algorithm).

### 5.3. Sonar TDoA (Time Difference of Arrival) Following

In this approach, a combination of radio and ultrasonic sensors is used as a feasible alternative to person following [14]. Several methods have recently been proposed for determining the position of a mobile node by means of measuring radio signals—time of arrival (ToA), time difference of arrival (TDoA), angle of arrival (AoA), received signal strength (RSS) [19].

The developed system implements TDoA for the measurement of distances. For this purpose, we use ultrasound-based signals to measure the distance between two points, and radio signals for synchronization. For localization estimation, we use three measurement devices and the trilateration technique. To perform the measurements, we take advantage of the fact that for each kind of signal, we know their different propagation speeds, so we can measure the time difference of arrival. For further details of this technique, refer to previous work in [14].

The hardware devices included in this setup are: (i) Erratic Videre mobile platform [18], (ii) Hagisonic ultrasonic sensors (transmitters/receivers) [20], (iii) Standard 433 MHz radio transmitter/receiver modules [21] and (iv) Handy Board, a general purpose board based on the 68HC11 microcontroller, widely used in the field of mobile robots for educational, hobbyist, and industrial purposes [22].

**Figure 10.**
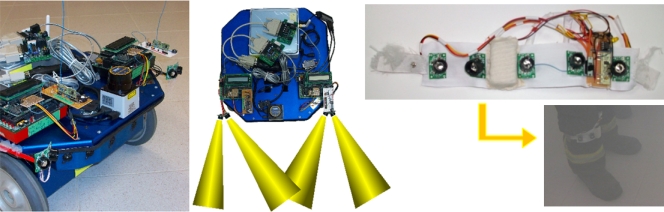
Erratic Videre mobile platform equipped with 2 measurement boards based on TDoA (left). Ultrasonic beam patterns (center). Sonar elastic ring, suitable to be attached to a person leg (right).

With this configuration (see Figure 10), the robot can estimate precisely the position of the person to be followed. The human carries a transmitter ring of ultrasound sensors (see Figure 10 right), following the same principle described above.

## 6. Comparative Results

In this section, the results obtained from the experiments made using the three methods described above (Sonar Ring following, Laser Range Finder following and Sonar TDoA) are discussed. As mentioned before, a squad composed of a human and two robots is used in the experiment. The objective of the first robot is to maintain a constant speed and an accurate trajectory along the path, in order to properly reproduce the conditions of the experiment. To compare the performance of each method, 5 trials at different 4 velocities has been done for each following method.

The results for the Laser and Sonar Ring methods can be seen in Table 1. Results for Sonar TDoA can be seen in Table 2 left. The table shows the percentage of following correctly achieved by the robot at different speeds. The human following is considered to be correctly achieved when the follower robot goes behind the human at constant speed and following the predefined trajectory. Speed of the person followed ranges from 0.10 to 0.25 m/s in steps of 0.05 m/s. These speeds are adequate for the robot platforms used in the experiments. At higher velocities, it is not easy to control the robots in order to follow the conditions of the experiment (constant speed and following the predefined trajectory), and it is not possible to compare the three proposed methods, as their performance decreases significantly. The percentage achieved shows how many curves the robot has been able to properly carry out. A percentage of 100% indicates that the follower robot has been able to follow the human during the entire predefined trajectory. The percentages of 25%, 50% and 75% indicate that the follower robot has lost the human after the first, second and third curve respectively. A percentage of 0% indicates that the follower robot has not been able to follow the human from the beginning.

Table 1 left shows that the performance of the person following using just the built-in Sonar Ring is not very reliable in general. Only at 0.10 m/s, the robot has been able to complete the full path once. The robot loses the tracking objective at curves. This result can be explained by the smaller precision obtained in general with the sonar ring sensors with respect to other more precise like the laser rangefinders.

In Table 1 right, we can see the results for the Laser Range Finder following method. In this case the performance is better even at higher speeds. The robot can follow the person even in the most difficult curve at the minimum speed of 0.10 m/s. At higher speeds, the robot loses the tracking objective at curves. This result can be explained by the better precision, resolution and number of samples per second obtained from the laser sensor.

Finally, in Table 2 left, we can see the results for Sonar TDoA. In this case the performance is quite good at higher speeds. The reason is that the robot can estimate precisely the position of the person to be followed thanks to the transmitter ring of ultrasound sensors (see Figure 10 right). Furthermore, the advantage of this method is that it does not need an environment with good visibility and thus can work even in smoky conditions.

**Figure 11.**
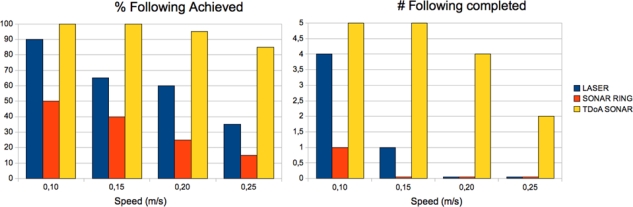
Comparative Results. % Follow. achieved (left). # Follow. completed (right).

Table 2 right summarizes the results of the three methods. The chart in Figure 11 summarizes also the percentage of following achieved (left), and the number of full trajectories completed (right).

## 7. Conclusions

This article presents a state-of-the-art of different methods traditionally used to perform person following with mobile robots. The problem has been solved, in most of the cases, by the use of machine vision. It is important to take into account that in some special circumstances, such as a smoky environment, the use of optical sensors is not a good solution. The proposal of this article is to use a system to determine the amount of smoke in the environment, and depending on the situation use a different combination of sensors to perform robot navigation an person following.

The use of ultrasound sensors is an alternative solution to LRF. Their performance when using a sonar ring is much lower than laser, but in some circumstances, like in smoky environments, they can provide a good solution. The proposed Sonar TDoA method, using a combination of radio and ultrasonic sensors is used as a feasible alternative to person following, and performs quite well even in smoky environments. The proposed smoke detection method using a video camera allows the robot to determine which combination of sensors to use in each situation, allowing the maximization of performance.

In future work, we are planning to extend the idea of multi-sensor detection and following of objects and persons, to underwater intervention environments, in the context of the FP7 TRIDENT project [23], where the low-visibility problem is also present.

## Figures and Tables

**Table 1.** Following experiments. **(Left)** Sonar Ring. **(Right)** Laser.

Speed (*m/s*)	% following achieved				Speed (*m/s*)	% following achieved			
	0.10	0.15	0.20	0.25		0.10	0.15	0.20	0.25
#1	100%	25%	25%	25%	#1	100%	100%	75%	50%
#2	50%	25%	25%	0%	#2	100%	50%	50%	25%
#3	25%	50%	25%	25%	#3	100%	75%	50%	50%
#4	25%	25%	25%	25%	#4	50%	50%	75%	25%
#5	50%	75%	25%	0%	#5	100%	50%	50%	25%
Average	50%	40%	25%	15%	Average	90%	65%	60%	35%
Completed	1	0	0	0	Completed	4	1	0	0

**Table 2.** **(Left)**TDoA Sonar following experiments. **(Right)** Global following comparative.

Speed (*m/s*)	% following achieved			
	0.10	0.15	0.20	0.25
#1	100%	100%	100%	75%
#2	100%	100%	100%	75%
#3	100%	100%	75%	100%
#4	100%	100%	100%	75%
#5	100%	100%	100%	100%
Average	100%	100%	95%	85%
Completed	5	5	4	2

Speed (*m/s*)	% following achieved (# completed)			
	0.10	0.15	0.20	0.25
Laser	90% (4)	65% (1)	60% (0)	35% (0)
Sonar Ring	50% (1)	40% (0)	25% (0)	15% (0)
TDoA Sonar	100% (5)	100% (5)	95% (4)	85% (2)
